# The relationships of school-based sexuality education, sexual knowledge and sexual behaviors—a study of 18,000 Chinese college students

**DOI:** 10.1186/s12978-017-0368-4

**Published:** 2017-08-25

**Authors:** Chunyan Li, Zixi Cheng, Taiwen Wu, Xiao Liang, Junjian Gaoshan, Lihe Li, Ping Hong, Kun Tang

**Affiliations:** 1 Department of Global Health, School of Public Health, Peking University, No.38 Xueyuan Road, Haidian District, Beijing, China 100191; 2 School of Basic Medical Sciences Peking University, Beijing, China; 3 China Family Planning Association, Beijing, China; 4 United Nations Population Fund China, Beijing, China

**Keywords:** Sexuality education, College students, Sexual knowledge, Sexual behavior

## Abstract

**Background:**

A growing prevalence of unexpected pregnancies and younger age of sexual debut is observed among Chinese young people, while they lack formal sexuality education from schools and parents. It is necessary to measure their knowledge level of sexual and reproductive health, and how such knowledge associates with their sexual behaviors and reproductive health outcomes, which would shed light on the effectiveness of sexuality education in China.

**Methods:**

An Internet-based questionnaire survey was conducted from January to August, 2015. 130 colleges were selected from eastern, central, and western parts China with a good balance of geographic distributions. The survey link was subsequently delivered to the focal points in each college for voluntary participation, targeting on undergraduates aged 18 ~ 25. Information on demographics, experience of school-based sexuality education (defined as any course introducing information on sexual and reproductive health) and SRH knowledge quiz was collected. Multivariate linear regression and logistic regression were applied to explore the relationship between students’ SRH knowledge, sexual behaviors and reproductive health outcomes, such as sexual intercourse (penetrative sex by vaginal or anal), unprotected sex, pregnancy and abortion, etc.

**Results:**

A total sample of 17,966 Chinese college students (mean age = 20.2, 60.4% female) eventually entered the analysis. Only 55.6% of the respondents self-reported having received sexuality education before, and they scored significantly higher (2.33/4.00) in the SRH knowledge quiz than those who had not (1.75/4.00). Among the sexually experienced students (*n* = 3639, 20.2%), both males and females with higher SRH knowledge were less likely to report having experience of (partner’s) pregnancy or abortion (OR < 1, *p* < 0.05). In the group of sexually experienced males, those with higher SRH knowledge had a slightly later age of sexual debut (coefficient = 0.28, *p* < 0.001), and were less likely to have unprotected sex during the last or in most sexual intercourses (OR = 0.82, 95%C.I.: 0.69 ~ 0.96).

**Conclusion:**

Students’ experience of school-based sexuality education may be positively associated with their SRH knowledge level as well as with their sexual behaviors and reproductive health outcomes, but such associations were stronger among males than females. A more effective implementation of school-based sexuality education needs to be scaled up, and a gender-sensitive education strategy to different needs is desirable for SRH promotion among Chinese young people.

## Plain English summary

With the aims to better understand the knowledge level of sexual and reproductive health (SRH) among Chinese youth and how this is associated with their sexual behaviors and reproductive health outcomes, this study conducted a series of quantitative analyses using the data from an Internet-based survey that investigated SRH among Chinese college students. We looked specifically into the associations of school-based sexuality education, knowledge on SRH with sexual behavior and reproductive health outcomes among college students. A total number of 17,966 undergraduates aged between 18 ~ 25, from over 130 Chinese colleges were included in the analyses. Results showed that only a half of the respondents reported having received school-based sexuality education, and they scored significantly higher in the SRH knowledge quiz. A higher SRH score was found to associate with better sexual behaviors and reproductive health outcomes. Students with higher level of knowledge on SRH were less likely to report negative reproductive health outcomes such as unintended pregnancy or abortion (in both males and females), and were more likely to use contraceptive methods in the last or the most sexual intercourses (only in males). Such findings support the need for a better implementation of school-based sexuality education in China, and for policy makers to employ a gender-sensitive approach, especially in empowering the girls, when designing education programs.

## Background

Even after decades of efforts on sexual and reproductive health (SRH) promotion from governments and organizations, the youth group aged between 15 ~ 24 is still facing many health challenges across the world [1, 2]. They account for 23% of the overall burden of disease due to unexpected pregnancies and childbirths [3], nearly one seventh of incidence of HIV infection [4]; among females aged 15 ~ 19, pregnancy-related death is the second leading cause of mortality [5]. Worldwide, certain designs of SRH intervention projects, such as school-based curriculum of sexuality education, training for parent-child communication or community-based intervention, have been proved to be considerably successful during the past years [6].

Nevertheless, the question whether sexuality education should adopt an abstinence-only strategy or provide “evidence-informed comprehensive sexuality education” have been debated for decades [7]. Experts, religious leaders and educational authorities have debated over the effectiveness and ethical rightness of different approaches; however, few high-quality research studies have shown the positive effect on sexual behavior change or the protective effect on negative SRH outcomes through the “abstinence only” approach, whereas the comprehensive sexuality education does make a significant achievement [7]. At the same time, such educational success could be jeopardized by the poor implementation and absence of evaluation in intervention projects [8].

In China, where a population as large as 227 million of young people aged 18 ~ 24 reside [9], the promotion of sexuality education from its Central Government could date back to as early as 1960s, when Premier Zhou Enlai officially declared the need to “popularize scientific sexual knowledge” among adolescents [10]. Sexuality education was formally introduced into school curriculum in 1988 and then has developed into an age-appropriate prevention strategy [11]. In the Guideline on HIV Prevention Education for Primary and Middle School Students issued by the Ministry of Education of China in 2008, all schools are required to “offer 6 to 7 hours of health education each semester”, “ensuring no less than 6 hours in the 3-year middle school education and 4 hours in the 3-year high school education are allocated to HIV prevention as well as sexual health education” [12]. The above policies, however, are widely considered to be inadequate for its insufficient class hours and lacking of teaching human resources [13], proven by the fact that only 19.8% of Chinese college students know the scientific names for male genital organs [14] . The lacking of formal sexuality education from schools or parents has resulted in that Chinese adolescents and young adults mostly obtain information about sex from the Internet [13], including false and violent ones. According to the data from the Chinese Health Statistics Yearbook published in recent years, there are over 6.5 million cases of abortion occurring every year [15], of which nearly a half are under 25 years old [16]. A meta-analysis of Chinese young people’s sexual behavior reported an estimated rate of unintended pregnancy and abortion among the sexually active youth group as 15.1% and 10.8%, respectively; as high as 53.6% of young people reported not using condoms during the last sexual intercourse [17].

The discordance between the effects of what the national sexuality education strategy was expected to achieve and the high prevalence of risky behaviors among young people in reality, makes it urgent for policy makers to evaluate the ongoing programs, as well as to identify the risky behavioral pattern among young people for future policy adjustment. So far, there is limited data about the relationship between SRH knowledge and sexual risk behaviors among Chinese students. The primary objective of this study was to understand how college students’ knowledge on SRH influences their behavioral pattern when they encounter sex, and what different reproductive health outcomes they would have experienced. In order to shed the light on the effectiveness of school-based sexuality education programs so far in China, the study also tried to explore the association between one’s reporting experience of receiving school-based sexuality education programs and his or her knowledge level on SRH.

## Methods

### Procedure

An Internet-based questionnaire survey was conducted from January to August in 2015 through a multi-stage sampling approach. A total number of 130 colleges were selected from eastern, central, and western China with a good balance of geographic distributions. The questionnaire was subsequently delivered to the focal points in each school. Each school then posted the survey link on social networks of that specific school for voluntary participation, as a way of convenience sampling. In order to raise the response rate, before starting answering the questionnaire, the participants were informed of having the chance to get a random amount of monetary remuneration (ranging from 1 ~ 200 RMB, and 200 RMB distributed in total) after successfully completing the questionnaire. The payment was directly made through the survey platform to individual participant. At the end, a total number of 10 participants were remunerated. To avoid repetitive answering from the same person, each IP address was restricted of only one chance to respond.

### Participants

The survey received 20,088 completed responses in total. Respondents who entered our final analyses were limited to (1) respondents agreed to the informed consent procedures, (2) Chinese young adults aged between 18 ~ 25, (3) undergraduate students who were enrolled in colleges within the Mainland of China at the time of survey, and (4) all questions were answered and submitted. After applying these inclusion criteria, individual data of 17,966 college students were used in the present analysis, of whom 90% came from the 130-targeted schools. The remaining 10% were from about 50 other schools, who happened to see the survey link posted in the targeted schools, hence answered the questionnaire. As this group of respondents fully met the inclusion criteria, we therefore included them in all our analyses.

### Questionnaire and variables

The questionnaire was developed independently by the research team. It is in Chinese (Mandarin) and composed of four parts with a total number of 52 questions, including demographic characteristics, SRH knowledge quiz and preferences over types of SRH education (e.g., peer education, online courses and brochures), gender and sexual relations and the final part, reproductive health outcomes and healthcare seeking behaviors. In this study, we focused on the data related to respondents’ experience of school-based sexuality education, knowledge level of SRH, sexual behaviors, reproductive health outcomes as well as demographic characteristics, and variables of interest were created accordingly.

One’s experience of sexuality education was measured by two aspects, i.e. whether and when they received such education. In the survey, students were asked whether they had received any form of school-based sexuality education (defined as “puberty education, reproductive health education or sexuality education” in the questionnaire, and the definition was placed right next to the question) during their school time. If respondents answered “yes”, 5 follow-up options of schooling stages were then presented as primary school, middle school, high school, the first two years of college and the later two years of college. Respondents were able to make multiple choices. In this study, the schooling stages of the last two were merged into one as “college”. Students’ SRH knowledge level were measured by number of correct answers to a short quiz (coded as SRH score, scaled 0 ~ 4). The quiz contained 8 questions in four dimensions, including knowledge on contraceptive use, HIV/AIDS, abortion/pregnancy and masturbation (Table 1).

**Table 1:** Items of SRH quiz from the survey questionnaire^a^

Dimension	Items (True or False)
1. Contraceptive Use	1.1 Withdrawal is an effective method of preventing pregnancy.
	1.2 Using condoms could 99.9% protect women from getting pregnant.
	1.3 Only married women could use Intra-Uterine Device (IUD).
2. HIV/AIDS	2.1 A person can get HIV by sharing drinking bottles or utensils with someone who lives with HIV.
	2.2 All pregnant women infected with HIV will have babies born with HIV.
3. Abortion/Pregnancy	3.1 Painless surgical abortion is safer than a regular surgical abortion^b^.
	3.2 A woman can get pregnant once she has sexual intercourse with a man.
4. Masturbation	4.1 Masturbation will damage one’s health.

^a^All statements were in Chinese (Mandarin) in the original questionnaire

^b^In China, due to commercial advertisement (especially from private clinics/hospitals), painless abortion is portrayed as a safer and convenient approach compared with traditional abortion, while actually the health outcomes of the two approaches are similar. These advertisements make some young people wrongly believe that unplanned pregnancy is not a big problem since there is such a ‘easy’ way to solve it, which then leads to a more careless attitude toward safe sex

Eight outcome variables were included in this study. “Sexual debut before the time of survey” was classified as “ever” versus “never” having had sexual intercourse before, and respondents were informed that “sexual intercourse” was specifically defined as penetrative sex either by vaginal or anal. Age at sexual debut and total number of past sex partners were coded as continuous variables. One’s first sex partner was dichotomized into “intimate” (defined as one’s boyfriend/girlfriend/wife/husband in the questionnaire) and “non-intimate”. Whether used contraception in the last sexual intercourse, whether used contraception in most sexual intercourses, whether experienced pregnancy ever (for male: being responsible for partner’s pregnancy) and whether experienced abortion ever (for male: being responsible for partner’s abortion) were dichotomized into “yes” and “no”. Besides, eight socio-demographic and behavioral covariates were used in the analysis. Age at survey was coded as a continuous variable. Monthly expenditure, hometown type, sexual orientation, family structure at the time of survey (1.nuclear/extended family:respondents living with both biological/adoptive parents, and with/without grandparent(s); 2.the other family structures except those mentioned in 1.), parents’ highest level of education, alcohol use and tobacco use were treated as categorical variables.

### Data analysis

Data was firstly extracted from the survey platform into Microsoft Excel, and then was converted and analyzed using Stata for Mac 12.1 (Statacorp, TX, USA). Descriptive statistics and regression analyses were applied in this study. All demographic variables were presented as frequencies and proportions in two gender groups. Multivariate-adjusted regression model was applied subsequently. First, subjects’ average SRH scores by experience of school-based sexuality education (adjusted for demographic characteristics) were calculated through the multivariate-adjusted linear regression model. In the second step, association analyses between one’s SRH scores and outcome variables were performed in two gender groups separately. To be specific, in this step, multivariate-adjusted logistic regression was performed for categorical outcome variables (whether had sexual debut before the time of survey, type of first sex partner, whether used contraception in the last/most sexual intercourse(s), ever pregnancy or ever abortion), and multivariate-adjusted linear regression was performed for continuous outcome variables (age of sexual debut, number of past sex partners). The covariates were included in all regression models. *Odds ratio* (OR) and linear regression coefficient were used as indicators for the extent of association between the exposure variable and outcome variables. The significance level set as two-tailed *p* < 0.05 in all data analyses.

## Results

### Sample characteristics

The total sample that eventually entered our analysis had a mean age of 20.2 years (SD = 1.2), and was 60.4% female. 2.5% had an homosexual orientation, and 5.1% had an bisexual orientation. Substantial variation existed in monthly expenditure, with 46.9% below 1000 RMB/month, 7.5% above 2000 RMB/month. The sample was predominantly students who grew up in the urban areas, and the vast majority were non-smokers (91.2%). Over half of the sample self-reported as occasional drinkers (< 10 times/year). Students from nuclear or extended families comprised of 89.5%. Middle school and high school were the two most prevalent education level regarded as the highest education of one’s parents (Table 2). For outcome variables (data not shown), close to 20.3% of the students reported having had sexual intercourse before. Among the sexually experienced students (*n* = 3639), 85.9% had one’s intimate partner as the first sex partner; 23.8% reported a sexual debut age of younger than 18 years old (24.6% in males and 22.9% in females); 83.6% reported using contraceptive methods during their last sexual intercourse, and 94.6% used contraceptive methods in most of their sexual intercourses. A relatively small number of female respondents had experienced pregnancy (11.0% in total sexually experienced females) or abortion (9.9% in total sexually experienced females).

**Table 2:** Basic social and demographic characteristics of the study population by sex

Variables	Male		Female		Total	
	n	%	n	%	n	%
	7118	39	10,848	62	17,966	100
Age						
≤ 19	1879	26	3621	33	5500	31
20 ~ 21	4074	57	6023	56	10,097	56
≥ 22	1165	16	1204	11	2369	13
Monthly expenditure						
< 1000	3149	44	5729	53	8878	49
1000 ~ 2000	3367	47	4375	40	7742	43
> 2000	602	8	744	7	1346	7
Alcohol						
non-drinker	922	13	4435	41	5357	30
occasional drinker	3620	51	5642	52	9262	52
infrequent drinker	2235	31	700	6	2935	16
frequent drinker	341	5	71	1	412	2
Tobacco						
never	5724	80	10,655	98	16,379	91
1 ~ 10cigarretes/day	1106	16	161	1	1267	70
> 10cigarretes/day	288	4	32	0	320	2
Hometown						
urban	2923	41	4203	39	7126	40
suburban	2122	30	3472	32	5594	31
rural	2073	29	3173	29	5246	29
Family Type						
nuclear/extended family	6351	89	9729	90	16,080	90
other	767	11	1119	10	1886	10
Parents’ Education						
primary school and be	740	10	1115	10	1855	10
middle school	2174	31	3693	34	5867	33
high school	2243	32	3474	32	5717	32
vocational school and	1961	28	2566	24	4527	25
Sexual Orientation						
heterosexual	6428	90	9660	89	16,088	90
homosexual	291	4	158	1	449	2
bisexual	277	4	634	6	911	5
not sure	122	2	396	4	518	3

### Experience of sexuality education

**Fig. 1:**
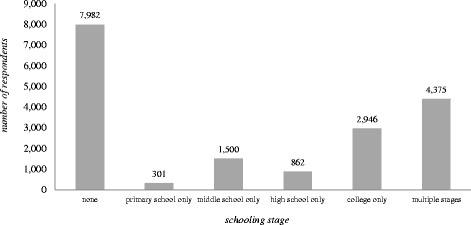
Distribution of schooling stages when students received sexuality education

**Fig. 2:**
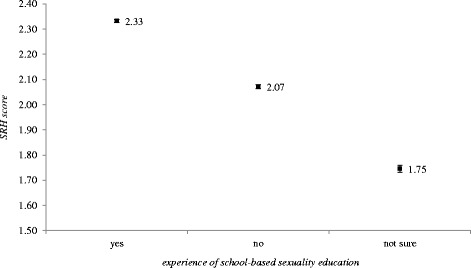
Average SRH scores and 95%C.I. by experience of school-based sexuality education

The data related to timing of receiving school-based sexuality education is based on respondents’ recalling experiences of “puberty education, reproductive health education or sexuality education”. Overall, 44.4%(*n* = 7982) students reported that they had never received any form of school-based education before, 1.7% (*n* = 301) only in primary school (*n* = 301, 1.7%), 8.3% (*n* = 1500) only in middle school, 4.8% (*n* = 862) only in high school, 16.4% (*n* = 2946) only in college, and 24.4% (*n* = 4375) received sexuality education in more than one schooling stages (Fig. 1). Among the students who received multiple-stage school-based sexuality education, 52.1%, 35.5% and 12.5% reported received sexuality education from two, three and four schooling stages, respectively (data not shown). Figure 2 shows the results of multivariate-adjusted average SRH scores by sexuality education experience, which indicates that students who reported having received school-based sexuality education before had a significantly higher SRH scores (means = 2.33, 95%C.I.: 2.33,2.34). For those who received sexuality education at multiple stages, an ascending tendency of SRH score could also be observed as the number of stages went up (data not shown).

### SRH scores and behaviors

Table 3 showed the logistic regression results, which was applied to explore the association between exposure and categorical outcome variables. For both the two gender groups, every one unit increase in the SRH score was associated with a slightly higher possibility to have experienced sexual debut before the time of survey (male: OR = 1.14, 95%C.I.:1.08 ~ 1.19; female: OR = 1.24, 95%C.I.: 1.18 ~ 1.30). The following analyses were conducted only in the sexually experienced group. Regarding the analysis of behavioral variables, the association between SRH score and the type of one’s first sex partner was found neither in males nor in females. Regarding contraception use, the results show that with every one unit increase in SRH score, male college students were less likely to have no contraception in the last sexual intercourse (OR = 0.87, 95%C.I.: 0.79 ~ 0.96), as well as less likely to have no contraception in the most sexual intercourses (OR = 0.82, 95%C.I.: 0.69 ~ 0.96). However, no such significance was found in females. Regarding the analysis of reproductive health outcomes, a higher SRH score was found negatively associated with one’s (partner’s) experience of pregnancy (male: OR = 0.87, 95%C.I.: 0.77 ~ 0.98; female: OR = 0.82, 95%C.I.:0.72 ~ 0.94) or abortion (male: OR = 0.83, 95%C.I. 0.73 ~ 0.94; female: OR = 0.84, 95%C.I.: 0.73 ~ 0.96) in both gender groups.

**Table 3:** The OR of having a certain kind of sexual behavior associated with 1-score increased in SRH knowledge by sex

Behaviors	Male		Female	
	OR	95% C.I.	OR	95% C.I.
*for all respondents (male = 7118, female = 10,848)*				
sex debut before the time of survey	1.14*	(1.08, 1.19)	1.24*	(1.18,1.30)
*for sex-experienced respondents (male = 2021, female = 1618)*				
1.First sex partner is non-intimate	0.94	(0.85, 1.05)	0.93	(0.80, 1.09)
2.No contraception in the last sex	0.87*	(0.79, 0.96)	0.93	(0.82, 1.05)
3.No contraception in most sex	0.82*	(0.69, 0.96)	0.82	(0.64, 1.05)
4. Ever pregnancy	0.87*	(0.77, 0.98)	0.82*	(0.72, 0.94)
5.Ever abortion	0.83*	(0.73, 0.94)	0.84*	(0.73, 0.96)

Note: *p < 0.05. Here adjusted for age, monthly expenditure, alcohol use, tobacco use, hometown, family structure, parents’ education and sexual orientation

Linear regression model was applied to the continuous outcome variables with all social-demographic and behavioral covariates adjusted. It is interesting to note that the two gender groups showed completely different results in this model, for all two outcome variables were significantly associated with the SRH scores in males, while no statistical significant association was found with any outcome in females (Table 4). Among sexually experienced male, respondents with higher SRH scores were more likely to have their sexual debut at a later age (coefficient: 0.28, *p* < 0.001), and more likely to have less past sex partners (coefficient: −0.04, *p* < 0.05).

**Table 4:** Linear regression coefficients assessing the association between SRH knowledge and sexual behavior by sex

	Male		Female	
	*β*	*p*	*β*	*p*
Age at sex debut	0.28	<0.001	0.02	0.566
Total number of past sex partners	−0.04	0.001	−0.01	0.339

Note: Adjusted for age, monthly expenditure, alcohol use, tobacco use, hometown, family structure, parents’ education and sexual orientation

## Discussion

### Main findings

Overall, nearly half of the respondents in this survey reported having never received school-based sexuality education before. For the other half, sexuality education was mainly conducted during middle school and college years. Significantly, sexuality-educated respondents scored higher in the SRH knowledge quiz than the others. Students with higher SRH knowledge had a slightly higher tendency to report having had sexual debut by the time of survey, but less likely to have experienced (partner’s) pregnancy, or (partner’s) abortion. Moreover, among the sexually experienced male respondents, those with higher SRH knowledge reported a higher proportion of contraceptive use in the last sexual intercourse or in most sexual intercourses, later age of sexual debut, but less total number of past sex partners. However, none of these indicators were found significantly associated with increase of SRH knowledge among the sexually experienced female respondents.

### School-based sexuality education and students’ SRH knowledge

Generally, respondents showed relatively limited access to SRH information for only half received school-based sexuality education ever. This proportion might to some degree be underestimated since the definition of sexuality education in the questionnaire only included “puberty education, reproductive health education or sexuality education”, failing to include HIV education as part of this education clearly. But it still indicates the failure in the uptake of sexuality education guidelines into routine practices in schools. Students who reported having received sexuality education before and received from more schooling stages, had higher mean SRH scores. To some degree, the positive impact of sexuality education on young people’s SRH knowledge that was found in the present study could buttress support for comprehensive sexuality education in China. Worldwide, it is more and more endorsed by a number of international organizations, such as United Nations Educational, Scientific, and Cultural Organization (UNESCO), that providing young people with the information and skills and starting as early as we can are crucial to help them make healthy and informed decisions about sex [18].

In China specifically, where this survey was conducted and where sexuality education has been written into its national education policy for decades, however, there is still a lack of an effectiveness evaluation system, neither is there incentive for the schools to ensure the stipulated class hours of health education. Many schools, regardless of stages, are appropriating the class hours of health education to other courses such as Mathematics or Chinese [19], in order to prepare the students for admission into better higher schools. According to the newest Chinese national guideline on health education (issued in 2008), sexuality education is designed as an age-appropriate approach that requires schools to provide different educational contents at different schooling levels, but emphasizing more on the topics of sex morality, self-esteem, reproductive system diseases, and warning the students of “the negative influence that pre-marital sex could bring about” [20]. Overall, China now tends to embrace an abstinence education strategy, which has been already proved ineffective in promoting SRH for the young people: according to an American study, between the two groups of heterosexual students of whom one received abstinence-only education while the other received comprehensive sexuality education, only the latter was found significantly less likely to report teen pregnancy (OR = 0.4, 95%C.I.: 0.22 ~ 0.69) [21]. The scope and content of sexuality education, as well as its implementation are still controversial issues in China; additionally, more longitudinal evidence is needed to evaluate its effectiveness in protecting Chinese students from risky sexual behaviors and negative reproductive health outcomes.

### Knowledge on SRH and sexual behavior patterns

In this study, students with higher SRH knowledge were more likely to report having sexual debut before the time of the survey. Although there is more evidence suggesting that sexuality education would delay sexual debut in the young people mainly through increasing their knowledge on SRH [4], still one very old study from the United States found similar results as in this one. In Oettinger’s analysis of data from the National Longitudinal Survey of Youth (1970s ~ 1980s), results indicated that enrolling in sex education might lead to “a higher hazard rate into sexual activity for females in this cohort”, “sex education in the 1970s probably had some causal influence on teen sexual behavior ” and “enabled teens to alter the risks of sexual activity” [22]. In the multivariable-adjusted regression model, we found that students who had a higher level of SRH knowledge were generally older, which made it possible that they were more likely to experience sexual debut before the time of the survey. Also, students with higher SRH might be more willing to disclose such information. Another explanation for the present results could be the ongoing “sexual liberation” in current China. One study in college students from the Western China found a significant correlation between self-judgment of sexual liberation and leisure consumption (coefficient = 0.101), and the latter was significantly correlated to the number of sex partners (coefficient = 0.181) [23]. Growing up in a relatively conservative society on the topic of sex and reproductive health, such as China, young people could be more curious to experience sex once they learn something about “sex”.

Regarding reproductive health outcomes, negative associations between SRH knowledge and experience of (partner’s) pregnancy or abortion was found in both gender groups. Previous data analyses showed that repeated induced abortions were as high as 55.9% among Chinese women who had experience of induced abortion [16], and that unintended pregnancy was highly associated with lack of awareness of contraception [24]. Among the sexually experienced respondents in our survey, males with higher SRH knowledge reported a later age of sexual debut on average by the time of survey, and were more likely to use contraceptive methods in the last or in most sexual intercourses; but no significant results were found in females.

It needs to be pointed out that the sexual behavior of females was less sensitive to the increase of SRH knowledge in the present study. This could be explained by the power imbalances between male and female in sexual decision-making [25]. Females in most developing countries’ settings usually have a more limited access to information related to SRH than their male counterparts, which leads to less favorable outcomes of sexuality education. Moreover, a randomized control trial in Tanzania indicated that the *MEMA kwa Vijana* intervention, which combined in-school education, community promotion and youth-friendly health services into one program, reduced the number of sexual partners significantly only among boys even when girls received the same intervention [26].

In China specifically, such a difference might be due to the traditional Chinese sexual double-standard: in the male dominant society, males are more powerful in deciding their sexual activities; on the contrary, female sexuality is controlled more tightly [27] and still there are popular folktales indicating that women should be loyal to their first and only husband, or how a man would destroy himself if being enticed by a woman. This gender difference in the present results reflects in some degree the gender inequality. Therefore, one suggestion for sexuality education programs in China is to provide young people correct and comprehensive information on the use of contraceptive methods to prevent unintended pregnancy and abortion, to build their confidence in talking about sex and to access contraceptive methods without consideration of social stigma. It is also important to improve females’ negotiation skills in family planning discussions with their partners, while at the same time educating the males about power balance. A comparative review of two types of sexuality education intervention programs, one containing gender equality or balance while the other not, has found that the former one was much more effective in decreasing the rate of unintended pregnancy and sexually transmitted diseases [28]. Similar to many other developing areas, China should also include the gender equality, especially related to the area of SRH promotion, to its health education strategy. While recognizing the importance of school-based sexuality education, other social supports, such as ensuring access to youth-friendly health services for young people and campaigns to promote gender equality in college communities, are also essential to make a real change in reproductive health promotion among university students.

### Strengths & Limitations

The Internet-based survey covered more than Chinese 130 colleges, recruiting as many as eighteen thousand college students. Prior to this study, few studies focused on the relationship between sexuality education, sexual behavior and SRH knowledge level among young Chinese students, partly due to the sexuality education traditionally being overlooked. This study, however, included many sensitive questions in the survey, such as sexual experience, age at sexual debut, total number of past sex partners, and experience of pregnancy or abortion. Meanwhile, the Internet-based approach is more suitable for such a survey of sensitive topics. This survey also had several potential limitations. Compared to the face-to-face interview, the Internet-based approach might be more vulnerable to response bias [29]. The researchers accordingly conducted a thorough logic check and regrouped continuous variables to reduce the impact of extreme values. Secondly, this survey was cross-sectional which could only suggest the correlation between experience of sexuality education, SRH knowledge, various sexual behaviors and health outcomes, rather than casual effects. Thirdly, all these dependent variables were mainly based on respondents’ recall of experience, which may be vulnerable to recall bias.

## Conclusion

One’s experience of school-based sexuality education may have a positive association with his or her SRH knowledge level. Generally, a lower degree of SRH knowledge was associated with a higher prevalence of sexual risk behaviors such as unprotected sex, unintended pregnancy and abortion. As in other contexts, the SRH improvement in males was more sensitive to the increase in SRH knowledge. On one hand, the present study suggests a need for the Chinese educational authority to ensure the provision of school-based sexuality education programs implemented in different schooling stages, offering SRH information to students and protecting them from negative reproductive health outcomes. On the other hand, a gender-sensitive approach of sexuality education should be more emphasized, including gender equity education and a focus on empowering women in the discussions on contraception with partners.

## Abbreviations

95% C.I.: 95% Confidence Interval
HIV: Human immunodeficiency virus
OR: Odds ratio
SD: Standard deviation
SRH: Sexual and reproductive health
